# Tumor Targeting by *Fusobacterium nucleatum*: A Pilot Study and Future Perspectives

**DOI:** 10.3389/fcimb.2017.00295

**Published:** 2017-06-30

**Authors:** Jawad Abed, Naseem Maalouf, Lishay Parhi, Stella Chaushu, Ofer Mandelboim, Gilad Bachrach

**Affiliations:** ^1^The Institute of Dental Sciences, The Hebrew University-Hadassah School of Dental MedicineJerusalem, Israel; ^2^Department of Orthodontics, The Hebrew University-Hadassah School of Dental MedicineJerusalem, Israel; ^3^The Lautenberg Center of General and Tumor Immunology, The Hebrew University Hadassah Medical School, Institute for Medical Research Israel-Canada (IMRIC)Jerusalem, Israel

**Keywords:** *Fusobacterium nucleatum*, Gal-GalNAc, adenocarcinoma, bacterioncology, cancer

## Abstract

Colorectal adenocarcinoma (CRC) is a common tumor with high mortality rates. Interestingly, CRC was found to be colonized by the oral anaerobic bacteria *Fusobacterium nucleatum*, which accelerates tumor progression and enables immune evasion. The CRC-specific colonization by fusobacteria is mediated through the recognition of tumor displayed Gal-GalNAc moieties by the fusobacterial Fap2 Gal-GalNAc lectin. Here, we show high Gal-GalNAc levels in additional adenocarcinomas including those found in the stomach, prostate, ovary, colon, uterus, pancreas, breast, lung, and esophagus. This observation coincides with recent reports that found fusobacterial DNA in some of these tumors. Given the tumorigenic role of fusobacteria and its immune evasion properties, we suggest that fusobacterial elimination might improve treatment outcome of the above tumors. Furthermore, as fusobacteria appears to specifically home-in to Gal-GalNAc—displaying tumors, it might be engineered as a platform for treating CRC and the above common, lethal, adenocarcinomas.

## Introduction

It is estimated that about 20% of cancer incidence are linked to infectious agents (zur Hausen, 2009; Plummer et al., 2016). In contrast to the numerous known onco-viruses, *Helicobacter pylori*, the causative agent of gastric cancer is, to date, the only bacteria classified as a carcinogen (Plummer et al., 2016). Recently, the oral bacteria, *Fusobacterium nucleatum* was shown to accelerate the progression of colon cancer and to confer the growing tumor with protection against attacking immune cell (Kostic et al., 2013; Rubinstein et al., 2013; Gur et al., 2015a,b; Yang et al., 2017).

*F. nucleatum* is a gram negative oral anaerobe that plays a key role in the development of the dental plaque by physically bridging between early and late oral bacterial colonizers (Kolenbrander and London, 1993). *F. nucleatum* numbers rise 10,000-fold in the gingival inflammation that precedes periodontal disease (Moore and Moore, 1994; Socransky et al., 1998). *F. nucleatum* is also frequently isolated (often as pure cultures) from samples collected in preterm births (Hill, 1998; Han, 2011). Most recently, *F. nucleatum* was found to be enriched in colorectal cancer (Castellarin et al., 2012; Kostic et al., 2012).

Colorectal cancer (CRC) is the second most commonly occurring cancer, and the fourth most common cause of cancer death (Siegel et al., 2012). Thus, new approaches for CRC diagnosis and treatment are required.

It is assumed that transient bacteremia (frequent during periodontal disease) enables the trafficking of oral fusobacteria to CRC. Tumor-induced angiogenesis, increased blood-vessel permeability, hypoxia, and local immunosuppression, are non-specific factors that aid CRC colonization by blood-borne oral fusobacteria (Abed et al., 2016). However, CRC-specific recognition by fusobacteria, is mediated by the fusobacterial Fap2 lectin, that specifically recognizes and binds tumor-displayed D-galactose-β(1-3)-N-acetyl-D-galactosamine (Gal-GalNAc) (Yang and Shamsuddin, 1996; Abed et al., 2016). High Gal-GalNAc levels were also detected in CRC metastases and were correlated with fusobacterial gDNA occurrence in these metastases (Abed et al., 2016), demonstrating the ability of fusobacteria to colonize CRC metastases.

Once in the tumor, fusobacteria can accelerate cancer development by enhancing cellular proliferation (Rubinstein et al., 2013; Chen et al., 2017; Yang et al., 2017), creating a tumor-favorable inflammatory environment (Kostic et al., 2013) and by protecting tumors from killing by NK cells and tumor infiltrating T cells. The latter is mediated through activation of the TIGIT inhibitory receptor, by the fusobacterial Fap2 protein (in a Gal-GalNAc-independent manner) (Gur et al., 2015a). Not surprisingly, high fusobacterial abundance in CRC was correlated with poor disease outcome (Flanagan et al., 2014), suggesting that therapeutic elimination of CRC-fusobacteria should be considered.

Interestingly, due to their Gal-GalNAc—specific homing, *F. nucleatum* could potentially be used as a platform for specific targeting and elimination of Gal-GalNAc displaying tumors and metastases. In this regards, besides CRC, additional tumors were previously found to display Gal-GalNAc (Springer, 1984; Lin et al., 2011). Here, we therefore re-screened for tumors that display high levels of Gal-GalNAc.

## Materials and methods

### Tumor, and normal tissue samples

Cancer tissue microarrays MC5003b, MC2082a, and BN1002b were obtained from US Biomax inc. Details regarding each sample on the arrays are available on the US Biomax Inc. website.

### Gal-GalNAc quantification

Gal-GalNAc detection and quantification was performed as described previously (Abed et al., 2016). Briefly, the microarrays were blocked with PBS supplemented with 10% BSA, 10% FBS and 0.5% Triton for 2 h at room temperature followed by incubation with FITC-labeled PNA (Sigma-Aldrich, cat. No. L7381) (50 μg/ml in PBS) overnight at 4°C. The slides were then washed three times with PBS for 10 min each, and then incubated with Hoechst 33258 (Sigma-Aldrich, cat. No.94403) diluted 1:5,000 for 15 min at room temperature.

Fluorescence intensity of the sample-bound FITC-labeled PNA was evaluated using the ImagePro Analyzer 7.0 software (Cybernetics, USA).

## Results

### Elevated Gal-GalNAc levels are detected in adenocarcinomas

**Figure 1:**
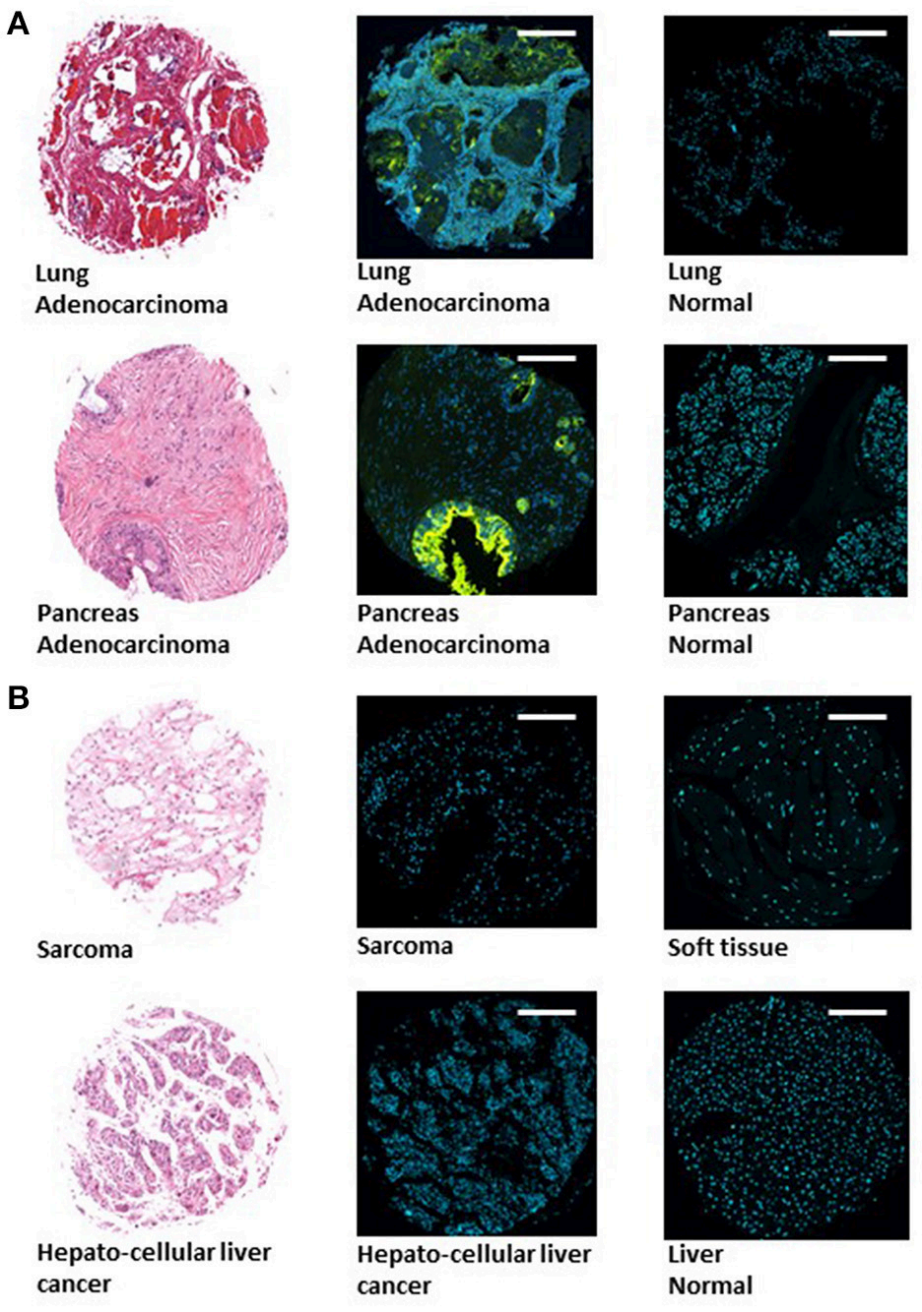
Images of representative tumors displaying high and low Gal-GalNAc levels. Tissue microarray (TMA) (Boimax inc.: MC5003b, MC2082a, BN1002b) were used to quantify Gal-GalNAc in tumor and matching normal control sections. Lung (top) and pancreas (bottom) adenocarcinomas displaying high Gal-GalNAc levels are presented in **(A)**. Sarcoma (top) and hepatocellular liver cancer (bottom) non-adenocarcinoma tumors displaying low Gal-GalNAc levels are shown in **(B)**. Left panels present H&E staining. Middle and right panels present FITC-labeled Gal-GalNAc-specific PNA (green) and Hoechst dye (blue) of tumor (middle panel) and normal (right panel). Bars shown are 250 μm scale.

Tissue microarrays (TMAs) (Boimax inc. MC5003b, MC2082a, and BN1002b) that contain samples of 20 different types of tumors (and their matching normal control tissues), were screened for Gal-GalNAc levels using a fluorescently labeled peanut agglutinin (PNA), a Gal-GalNAc—specific lectin (Abed et al., 2016). Representative images of sections of tumors that display high Gal-GalNAc levels (lung and pancreas adenocarcinomas) and of their matching controls (that display low Gal-GalNAc levels) can be seen in Figure 1A. Images of representative tumors that display low Gal-GalNAc levels are presented in Figure 1B.

**Figure 2:**
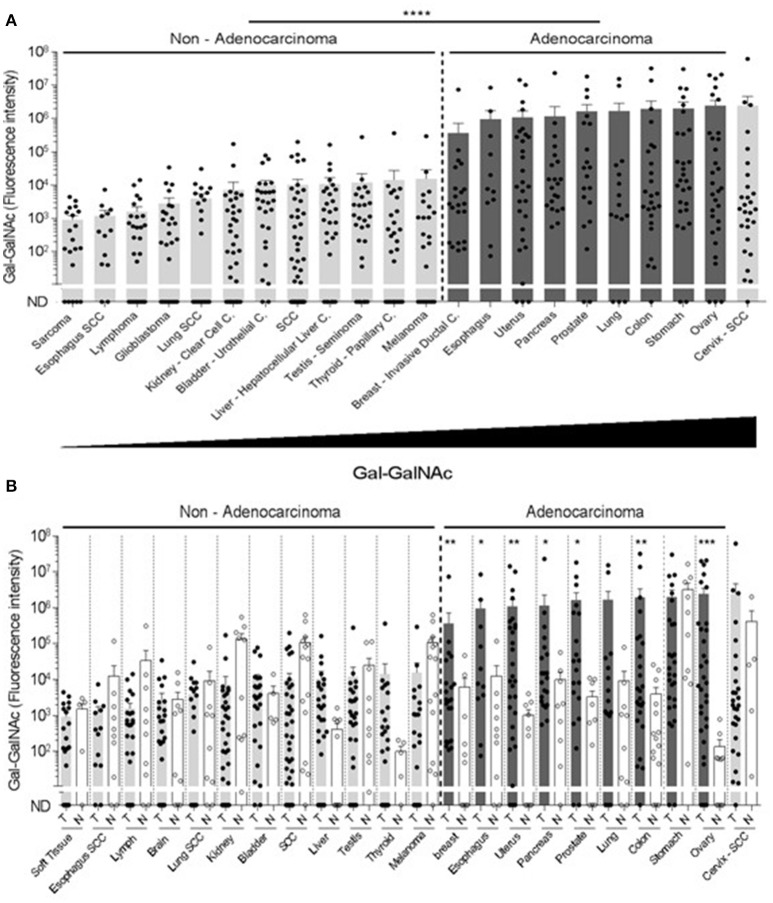
High Gal-GalNAc levels are displayed in human adenocarcinomas. **(A)** Tumors were arranged according to increasing Gal-GalNAc levels. As can be seen, all examined adenocarcinomas (dark gray) displayed high levels of Gal-GalNAc. **(B)** Gal-GalNAc levels in the tumors (closed symbols) described in **(A)** were compared to those in the matching normal tissue controls (open symbols). As can be seen, Gal-GalNAc levels in 7 out of the 9 presented adenocarcinomas were statistically significantly higher than those measured in the matching normal control tissues The normal tissue controls for esophagus, lung and skin were used twice for the respective esophagus adenocarcinoma and squamous cell carcinoma (SCC): the respective lung adenocarcinoma and SCC, and for the melanoma and SCC. Each symbol represents the fluorescent intensity of a different sample. Error bars indicate mean ± SEM. ^*^p < 0.05, ^**^p < 0.01, ^***^p = 0.0001 Two-tailed Mann-Whitney test. ^****^p < 0.0001 using two-tailed *t*-test.

Next, the examined cancers were arranged according to their Gal-GalNAc levels (Figure 2A). High Gal-GalNAc levels were detected in 10 types of tumors out of the 20 tested (Figure 2A). These tumors were of epithelial tissue with glandular origin or/and glandular characteristics, 9 of them adenocarcinomas (of stomach, prostate, ovary, colon, uterus, pancreas, breast, lung, and esophagus) and one a squamous cell carcinoma of the cervix. The Gal-GalNAc levels in 8 of these tumors, were higher than those in the matching normal tissue controls, 7 of them (all adenocarcinomas) with statistical significance (Figure 2B). The Gal-GalNAc levels in the stomach and cervix normal control samples were high and similar to those in the respective cancers. Conversely, in the non-adenocarcinoma tumors, Gal-GalNAc levels were similar to those in the matching normal tissue controls (Figure 2B).

### Occurrence of *Fusobacterium nucleatum* in tumors with high Gal-GalNAc levels

Interestingly and in agreement with our predictive results that fusobacteria can home-in and accumulate in high Gal-GalNAc displaying cancers, fusobacterial DNA were reported to be overabundant in pancreas (Mitsuhashi et al., 2015), breast (Hieken et al., 2016), and esophagus (Yamamura et al., 2016) adenocarcinomas and in normal and cancer stomach samples (Dicksved et al., 2009; Nardone and Compare, 2015). This, in addition to the well-known prevalence of fusobacteria in the high Gal-GalNAc -levels displaying colon cancer (Castellarin et al., 2012; Kostic et al., 2012; Abed et al., 2016).

## Discussion and future perspectives

The results above support our hypothesis that in addition to CRC, fusobacteria home-to and colonize additional tumors that display high levels of Gal-GalNAc. As *F. nucleatum* was shown to accelerate tumor progression (Kostic et al., 2013; Rubinstein et al., 2013; Gur et al., 2015a; Yang et al., 2017), fusobacterial elimination in these tumors might improve treatment outcome.

Numbers of periodontal bacteria, including fusobacteria, greatly increase during periodontal inflammation (Socransky et al., 1998; Hajishengallis et al., 2011). This raise in bacterial numbers together with the frequent bleeding of the gums during periodontitis, increases the probability of hematogenous translocation of oral bacteria to distant tumors. Indeed, as part of the growing interest in the effect of oral health on general health (Pihlstrom et al., 2005; Rautemaa et al., 2007), the relationship between periodontitis and cancer development is of growing interest (Hiraki et al., 2008; Zeng et al., 2016).

As fusobacteria appear to specifically home-to tumors and metastases displaying high amounts of Gal-GalNAc, fusobacteria might be used in the future as a platform for directing treatment (immunological or chemically based) to such cancers. It should be noted that not all of the samples of each type of adenocarcinoma displayed high Gal-GalNAc levels. This implies that a potential fusobacterial-based cancer therapy will have to be personalized to high Gal-GaNAc displaying tumors.

Bacterioncology, tumor-bacterial interactions, is a rapidly developing field. The tumor microbiome was found recently to play an important role in the effectiveness of cancer treatment (Bashiardes et al., 2017). Bladder cancer is routinely treated with the live bacterial tuberculosis vaccine Bacillus Calmette-Guerin (BCG) (Babjuk et al., 2011), and additional bacterial species are being explored for future cancer treatment (Quispe-Tintaya et al., 2013; Zheng et al., 2017).

It is interesting to note that the fusobacterial Fap2 surface protein, that mediates fusobacterial attachment to tumor-displayed Gal-GalNAc, also endows tumor protection by fusobacteria by activating the immune cells - suppressing TIGIT receptor. Both lectin and immunosuppression functions appear to be on different Fap2 epitopes. This is deduced from the fact that while tumor binding is inhibited by GalNAc (Abed et al., 2016), immunosuppression by TIGIT activation, is not (Gur et al., 2015a). It seems to make evolutionary sense to couple both tumor-associated traits on the same virulence factor.

As immunosuppression is undesired in a future fusobacterial-based tumor BacterioImmunotherapy, the challenging task of identifying and inactivation of the Fap2 TIGIT-activating domain remains.

## Author contributions

JA designed and carried out experiments, participated in writing the ms; LP, NM, and SC carried out experiments and participated in writing the ms; GB and OM designed experiments, and participated in writing ms.

### Conflict of interest statement

The authors declare that the research was conducted in the absence of any commercial or financial relationships that could be construed as a potential conflict of interest.
